# The trophic role of a large marine predator, the tiger shark *Galeocerdo cuvier*

**DOI:** 10.1038/s41598-017-07751-2

**Published:** 2017-08-09

**Authors:** Luciana C. Ferreira, Michele Thums, Michael R. Heithaus, Adam Barnett, Kátya G. Abrantes, Bonnie J. Holmes, Lara M. Zamora, Ashley J. Frisch, Julian G. Pepperell, Derek Burkholder, Jeremy Vaudo, Robert Nowicki, Jessica Meeuwig, Mark G. Meekan

**Affiliations:** 1 School of Biological Sciences and Oceans Institute, University of Western Australia, Crawley, WA 6009 Australia; 2 Australian Institute of Marine Science (M096), University of Western Australia, Crawley, WA 6009 Australia; 3 School of Environment, Arts, and Society, Florida International University, North Miami, FL 33181 USA; 4 College of Marine and Environmental Sciences, James Cook University, Cairns, QLD 4878 Australia; 5 School of Biological Sciences, University of Queensland, St Lucia, Queensland QLD 4072 Australia; 6 Institute for Marine and Antarctic Studies, University of Tasmania, Hobart, TAS 7001 Australia; 7 Reef HQ, Great Barrier Reef Marine Park Authority, Townsville, QLD 4810 Australia; 8 Australian Research Council Centre of Excellence for Coral Reef Studies, James Cook University, Townsville, QLD 4811 Australia; 9 Pepperell Research and Consulting Pty Ltd., Noosaville DC, Qld Australia; 10 The Guy Harvey Research Institute, Nova Southeastern University, Dania Beach, FL 33004 USA; 11 Elizabeth Moore International Center for Coral Reef Research and Restoration, Mote Marine Laboratory, 24244 Overseas Highway, Summerland Key, FL 33042 USA; 12 Centre for Marine Futures, University of Western Australia, Crawley, WA 6009 Australia

## Abstract

Tiger sharks were sampled off the western (Ningaloo Reef, Shark Bay) and eastern (the Great Barrier Reef; GBR, Queensland and New South Wales; NSW) coastlines of Australia. Multiple tissues were collected from each shark to investigate the effects of location, size and sex of sharks on δ^13^C and δ^15^N stable isotopes among these locations. Isotopic composition of sharks sampled in reef and seagrass habitats (Shark Bay, GBR) reflected seagrass-based food-webs, whereas at Ningaloo Reef analysis revealed a dietary transition between pelagic and seagrass food-webs. In temperate habitats off southern Queensland and NSW coasts, shark diets relied on pelagic food-webs. Tiger sharks occupied roles at the top of food-webs at Shark Bay and on the GBR, but not at Ningaloo Reef or off the coast of NSW. Composition of δ^13^C in tissues was influenced by body size and sex of sharks, in addition to residency and diet stability. This variability in stable isotopic composition of tissues is likely to be a result of adaptive foraging strategies that allow these sharks to exploit multiple shelf and offshore habitats. The trophic role of tiger sharks is therefore both context- and habitat-dependent, consistent with a generalist, opportunistic diet at the population level.

## Introduction

Marine megafauna such as large sharks, cetaceans, billfishes and tunas typically occupy upper trophic levels in marine food webs. Although their direct predation on and induced anti-predator behaviours (risk effects) in their potential prey can structure marine ecosystems^1–3^, it is often difficult to quantify their trophic interactions. First, top-order predators are a relatively rare component of food-webs and documenting their feeding behaviours within a three-dimensional environment presents some unique logistic obstacles that still challenge available technology^4^. Second, the prey targeted by many top-order species may not be consistent in space and time. Most predators can switch among potential prey taxa depending on context and availability in order to optimise energy gain from foraging behaviour^5^.

Difficulties involved in the direct observation of feeding behaviour of marine megafauna have led to the use of alternative techniques to provide insights into the process of foraging. One of the most common of these is the analysis of stable isotopes of carbon and nitrogen of consumers’ tissues, which can provide information on diet^6, 7^, trophic niche^8, 9^, trophic interactions among different species^10, 11^ and migratory movements^12, 13^. Ratios of nitrogen isotopes (^15^N/^14^N = δ^15^N) indicate the trophic position of a predator in the food web and the δ^15^N composition of its prey, whereas ratios of carbon (^13^C/^12^C = δ^13^C) indicate the basal source of carbon in the food chain^14^. Together, δ^15^N and δ^13^C can provide a general understanding of the structure of a food web^15^.

In marine ecosystems, values of δ^13^C in primary producers are indicative of environmental (inshore to offshore) and carbon source (benthic to pelagic) gradients^16, 17^. However, for highly mobile and slow-growing predators such as some large sharks, the interpretation of stable isotopic values must consider the time taken to incorporate isotopes into different tissues^7, 18^. Blood plasma and liver have rapid turnover rates due to their high catabolic rate and represent a short-term (days to weeks) integration of a predator’s diet^19^. In contrast, muscle, red blood cells, cartilage and fin have slower turnover rates and provide long-term (months to years) integration of dietary information^19, 20^. This means that the isotopic composition measured from different tissues within the same individual can resolve dietary information at different time scales^21^ and thus assess individual strategies of dietary specialisation^22^. Because stable isotopes integrate animal diets over time and space^23^, changes in composition in different tissues of mobile marine predators can “track” their use of isotopically distinct environments at different time scales^13^. This attribute has been used successfully to define shifts in diets of marine megafauna that relate to changes in use of habitats^24, 25^.

Here, stable isotope analyses were used to examine the trophic role of tiger sharks (*Galeocerdo cuvier*). These top-order predators are highly mobile, large-bodied sharks distributed globally in tropical, sub-tropical and temperate regions^26, 27^. Based on their diet, size and lack of predators, adult tiger sharks (>3 m) are classified as apex predators^28^. In at least some contexts, they play an important role in structuring the environments they inhabit^29, 30^. Tiger sharks have a diverse diet, feeding on prey from many trophic levels including invertebrates, teleosts, sea snakes, large marine herbivores such as sea turtles and sirenians, seabirds and other elasmobranchs^31–33^. Predation may even be replaced by facultative scavenging in situations where this behaviour offers greater food availability^34^. The species also exhibits high individual variability in movement patterns with some evidence of ontogenetic expansion in habitat utilisation^35–38^. It is likely, therefore, that their role in structuring marine ecosystems will vary both in space and with ontogenetic stage. Sampling over broad spatial and temporal scales and multiple habitats could allow incorporation of stable isotope data into studies of the spatial ecology (movement patterns, space use and habitat preferences) of these predators, and provide novel insights into both the motivations behind patterns (prey preference and foraging locations) and the role of these sharks within the various marine habitats that their movements encompass.

In this study, we compare the stable isotope composition of multiple tissues of tiger sharks collected at locations spread across their range in Australian waters. Sampling encompassed tropical and warm-temperate environments to provide insights into the trophic ecology and niche of the species across multiple habitats. This study also sought evidence for the influence of biological (size, sex) and spatial (latitude, location) factors that could influence the stable isotopic composition of tiger sharks and how that relationship changed at both short (days to weeks) and long (months to years) time scales by analysing tissues with different turnover rates.

## Results

**Figure 1:**
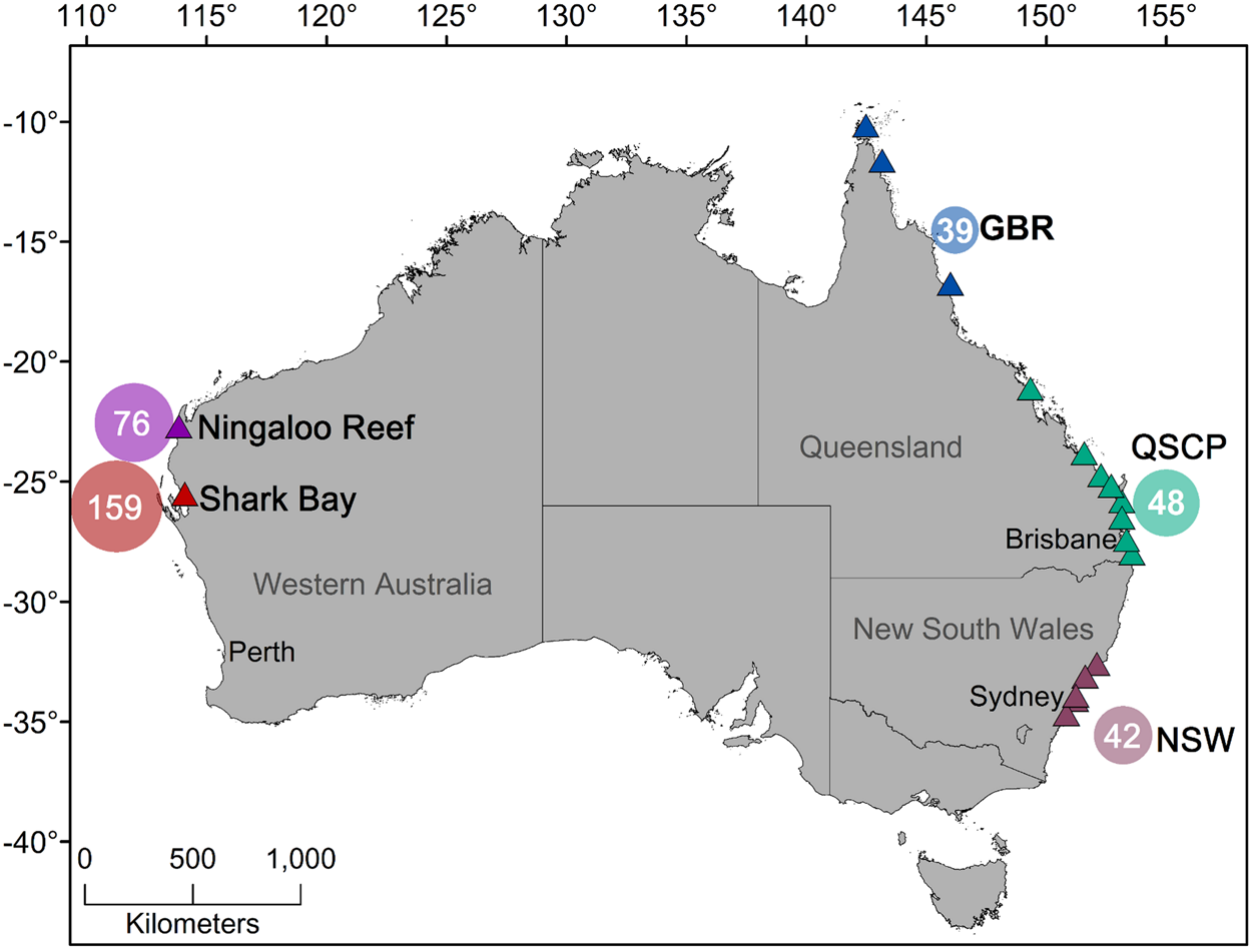
Map showing the study locations across Australia and sampling sites within each region in colour coded triangles. Circles represent relative and total sample size for each dataset. GBR = Great Barrier Reef, QSCP = Queensland Shark Control Program, NSW = New South Wales. Map was created with ArcGis 10.3 (http://www.esri.com/).

The dataset summed to 364 samples of multiple tissues (muscle, dermis, fin, whole blood, red blood cells) from 273 sharks collected at Ningaloo Reef, Shark Bay, Great Barrier Reef (GBR), Queensland (QSCP) and New South Wales (NSW) (Table 1, Fig. 1).

**Table 1:** Mean and standard deviation (SD) stable isotopic composition of tiger sharks sampled in the Queensland Shark Control Program (QSCP), New South Wales (NSW) the Great Barrier Reef (GBR) and Ningaloo and Shark Bay for each sex (F = female, M = male).

Location	Sex	N	TL	Muscle		Dermis		Fin		Red Blood Cells		Plasma	
				δ^15^N	δ^13^C	δ^15^N	δ^13^C	δ^15^N	δ^13^C	δ^15^N	δ^13^C	δ^15^N	δ^13^C
QSCP	F	36	207 ± 84.8	12.4 ± 1.0	−16.8 ± 0.7								
	M	12	161 ± 37.3	12.2 ± 1.1	−17.4 ± 0.5								
NSW	F	17	322 ± 50.9	12.6 ± 0.8	−18.2 ± 0.6								
	M	25	345 ± 39.5	12.3 ± 0.8	−18.3 ± 0.8								
GBR	F	14	296 ± 48.3	11.6 ± 0.9	−14.2 ± 1.6	11.3 ± 0.5	−12.0 ± 1.3			11.0 ± 0.3	−13.5 ± 1.9	11.5 ± 1.1	−13.4 ± 1.9
	M	2	232 ± 23.5	12.8 ± 0.7	−16.2 ± 0.1	11.9 ± 0.2	−13.8 ± 0.1			11.9 ± 0.5	−15.2 ± 1.1	12.3 ± 1.3	−15.7 ± 0.0
Ningaloo	F	20	348 ± 46.3			12.0 ± 0.4	−13.9 ± 1.4			11.6 ± 0.3	−16.6 ± 1.6	11.0 ± 0.5	−16.7 ± 1.2
Shark Bay	F	124	280 ± 59.2					11.8 ± 0.7	−16.6 ± 1.3	11.4 ± 0.5	−13.2 ± 1.6	11.6 ± 0.9	−15.0 ± 1.7
	M	23	268 ± 52.6					12.0 ± 0.6	−11.9 ± 1.4	11.0*	−13.1*	12.0 ± 1.9	−15.3 ± 1.7

δ^15^N and δ^13^C given in parts per mill (‰). N = number of samples, TL = mean total length (cm) ± SD.

*n = 1.

### Comparisons of stable isotopic composition among locations

**Figure 2:**
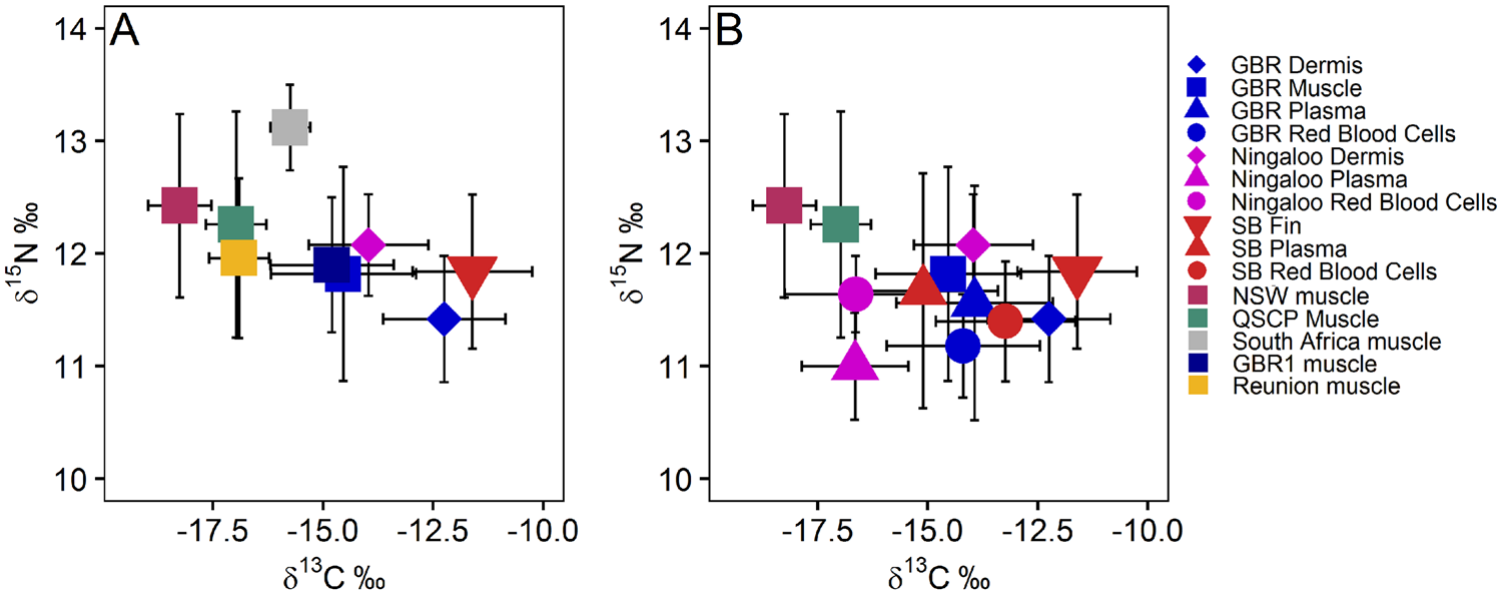
Mean and standard deviation stable isotopic composition of each tissue for each of the locations sampled (SB = Shark Bay, GBR = Great Barrier Reef, NSW = New South Wales, QSCP = Queensland Shark Control Program) for (**A**) slow tissues (muscle, dermis, fin) and (**B**) multiple tissues (muscle, dermis, fin, red blood cells, plasma), for sites where multiple tissues were collected. Data from GBR1, Reunion Island and South Africa obtained from published studies^39, 40, 68^.

A comparison of slow-turnover tissues (dermis, fin and muscle) showed a spread of stable isotope values that indicated variation in the ultimate sources of nutrition of tiger sharks across both latitude and shelf positions (inshore/offshore habitats) (Fig. 2A). Most of the variation occurred along the δ^13^C axis. Individuals from Shark Bay had higher δ^13^C, whereas sharks from the QSCP, NSW and Reunion Island^39^ had lower δ^13^C. Sharks sampled on the GBR had intermediate δ^13^C values and were very similar to those of muscle tissue collected and analysed by other studies in the same locality (Fig. 2A). However, δ^13^C values of dermis from GBR sharks were high and overlapped with those of Shark Bay individuals. There was limited variation in mean δ^15^N values of sharks among localities, with the exception of tiger sharks in South Africa^40^, which had higher δ^15^N values (Fig. 2A).

The collection of multiple tissues from the same animals in some localities allowed the isotopic composition of both fast (blood, plasma) and slow (muscle, dermis and fin) turnover tissues to be compared (Fig. 2B). Once again, there was greater variation in δ^13^C than δ^15^N values. Fast tissues tended to have lower δ^13^C compared to slow turnover tissues collected at the same locality. At Ningaloo, red blood cells and plasma had lower δ^13^C compared to dermis (Fig. 2B), whereas at Shark Bay, plasma had a lower δ^13^C compared to red blood cells and blood cells had lower δ^13^C compared to fin (Fig. 2B). Analyses of the GBR samples revealed a somewhat different pattern, with blood and plasma having lower δ^13^C compared to dermis, but muscle samples having similar δ^13^C values to these fast turnover tissues (Fig. 2B).

### Comparisons of stable isotopic compositions within locations

**Figure 3:**
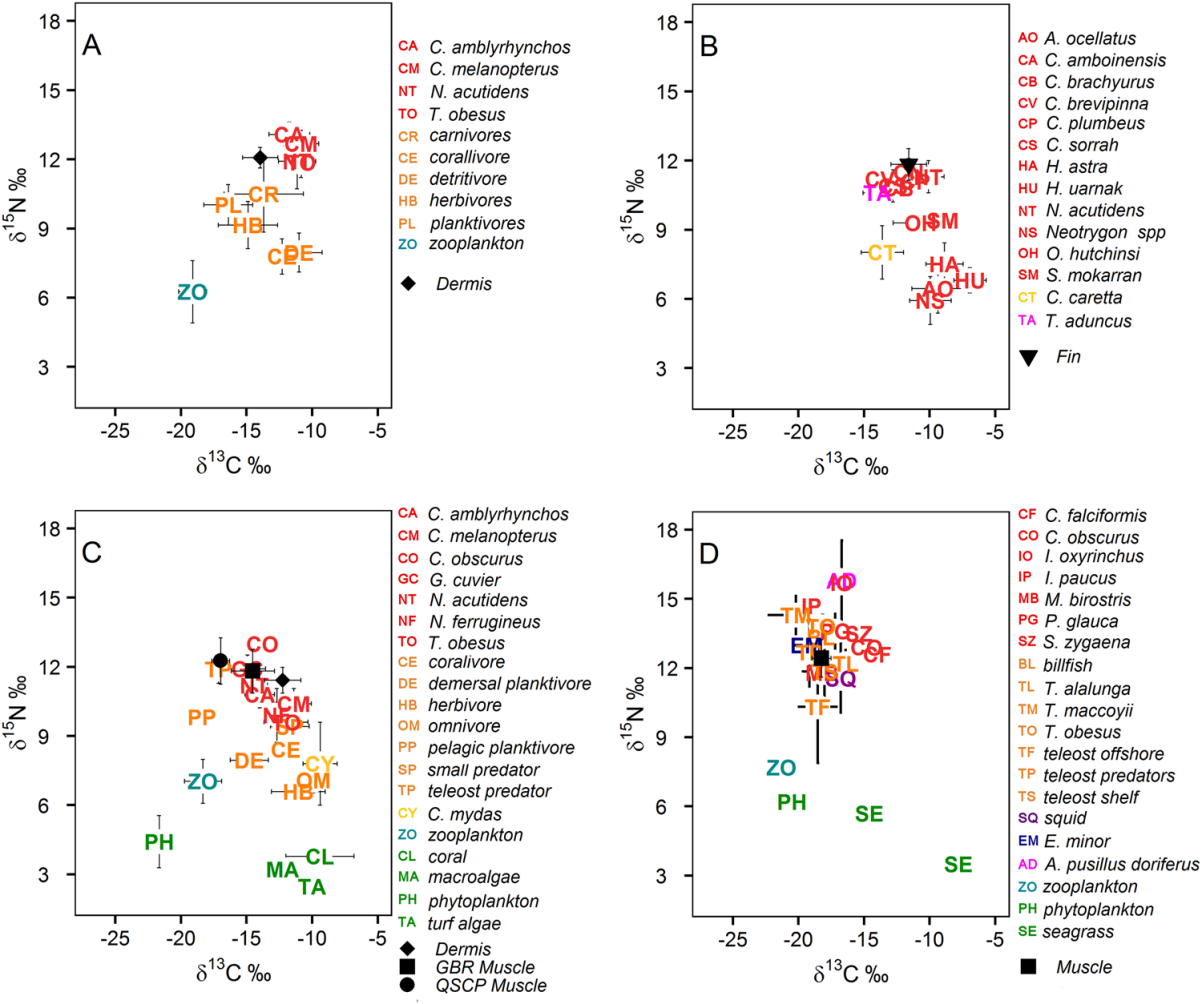
Mean isotope values (±SD) of slow turnover tissues (muscle, dermis, fin) of tiger sharks, other predators, consumers and primary producers at Ningaloo Reef, Shark Bay, Great Barrier Reef and New South Wales. Community data for each location was obtained from published studies^41, 55, 58, 61, 68, 86–90^ and L. Zamora (unpublished data). Red = elasmobranchs, orange = teleosts, yellow = sea turtles, magenta = marine mammals, purple = cephalopods, light blue = zooplankton, green = producers.

Stable isotopic composition of tiger sharks was plotted with those of other fishes, sharks and taxa from differing trophic levels at each location (Fig. 3). Only values for slow turnover tissues muscle and dermis of sharks were included in these plots since stable isotopic compositions for other vertebrates were largely derived from the analysis of muscle. At Ningaloo, dermal tissues of tiger sharks displayed lower average δ^15^N and δ^13^C values than reef sharks (Fig. 3A). In Shark Bay, tiger shark muscle had higher δ^15^N than all other sharks at this location (Fig. 3). On the GBR, tiger sharks had higher δ^15^N compared to other sharks at this location, with the exception of *C. obscurus*, the dusky shark. This pattern was consistent across tissue types (dermis and muscle) of sharks sampled on the GBR (Fig. 3B). Muscle tissue from sharks collected by the QSCP displayed similar δ^15^N values as dermis and muscle tissue from animals collected on the GBR, but had lower δ^13^C, suggesting a diet based on a more pelagic food chain for sharks sampled in the QSCP than on the GBR (Fig. 3C). The isotopic composition of muscle from tiger sharks collected on the NSW coast was also consistent with a diet based on pelagic food chains or different basal sources of carbon. In contrast to both the GBR and Shark Bay, the stable isotopic composition of Australian fur-seals (*Arctocephalus pusillus doriferus*) and a number of large pelagic fishes including tunas and mako sharks (*Isurus oxyrinchus*) had higher δ^15^N than those of tiger sharks, suggesting that these species feed at higher trophic levels. The isotopic composition of tiger shark tissues in this location was similar to those of billfishes (Fig. 3D).

### Comparison of tissues among locations

#### Muscle

**Figure 4:**
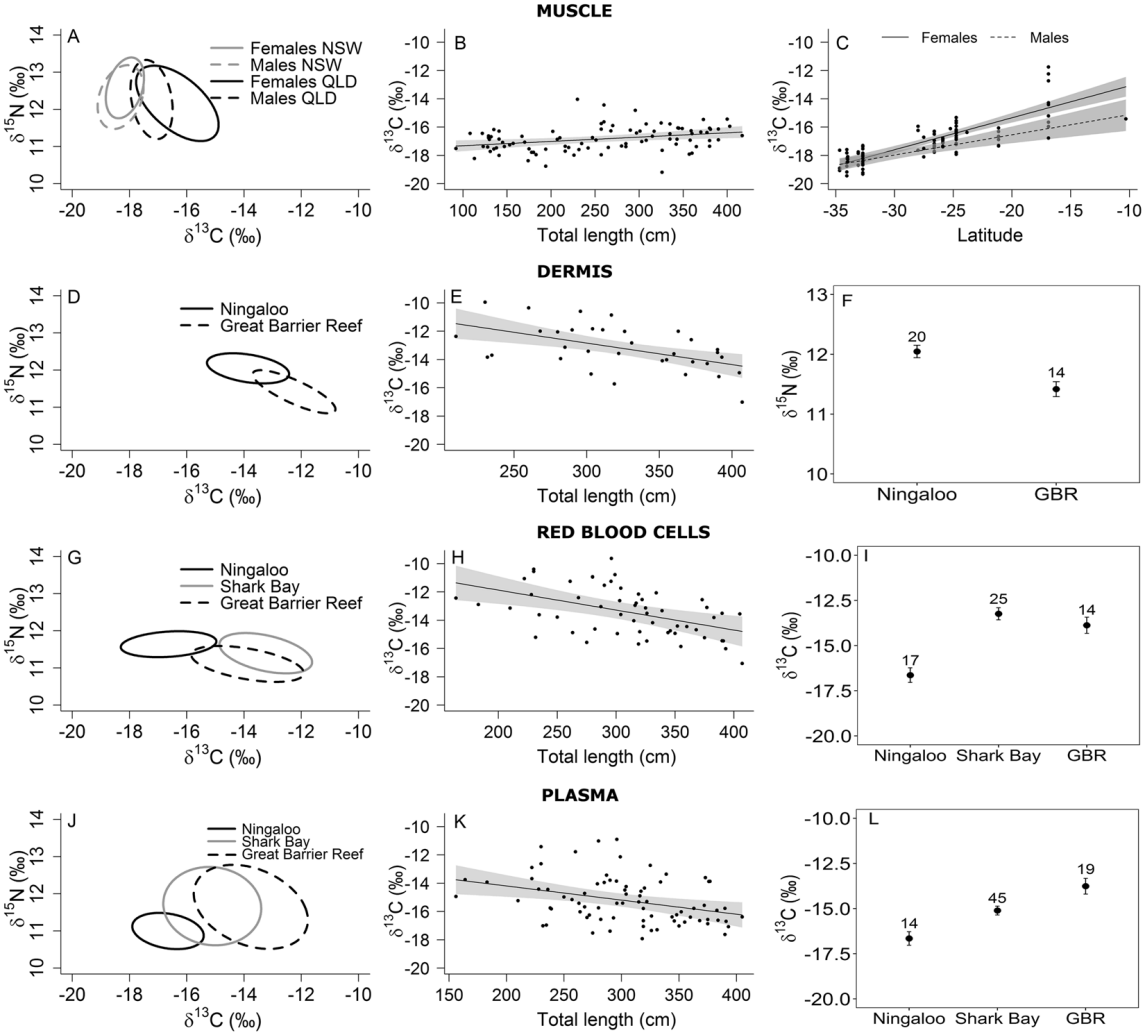
Muscle (**A–C**), dermis (**D–F**), red blood cells (**G–I**) and plasma (**J–L**). Standard ellipse areas for analysis of tissues corrected for sample size (SEAc) (**A,D,G,J**). Partial dependence plots of the relationship between δ^13^C and the explanatory variables in the top-ranked model, total length (**B,E,H,K**), the interaction between latitude and sex (**D**) and location (**F,I,L**). Points represent ﻿parti﻿al residuals and shaded areas show the 95% confidence intervals.

Differences in isotopic niches were assessed separately for each sex. For this analysis, muscle samples from the QSCP and the GBR were pooled (QLD; Fig. 4A–C). Female tiger sharks in QLD had a larger niche and higher trophic diversity than all other combinations of sex and location, based on the distribution of SEAb and TA (Table 2; Fig. 4A). The SEAc parameter for females from QLD showed that the ellipse for this group was stretched with dispersion both on the δ^13^C axis and the δ^15^N axis (E = 0.88, θ = −28.87°; Fig. 4A, Table 2). In contrast, male sharks in QLD waters had a greater dispersion over the δ^15^N axis (E = 0.77, θ = −72.85°) than the δ^13^C axis as did both males and females from NSW (E = 0.68 and 0.74, θ = 51.5 and 60.6, respectively). Sharks in NSW waters had a large overlap between sexes (0.68) and little difference in niche area (probability = 0.29). Females and males in QLD displayed high niche overlap (0.60), but little overlap with tiger sharks from NSW waters. Females in NSW showed the lowest trophic diversity and total area, despite having a larger SEAb than males in QLD (Table 2).

**Table 2:** Isotopic niche area described by Standard Ellipse Area (SEAc) (‰), Bayesian SEAb and parameters; N = number of samples, TA = total area of the convex hull, CD = mean centroid distance and standard deviation (SD), E = eccentricity, θ = angle of ellipse and CI = confidence interval. F = Females, M = Males, QLD = Queensland Shark Control Program and GBR, GBR = Great Barrier Reef and NSW = New South Wales.

Location	N	SEAc	SEAb	TA	CD ± SD	E	θ	95% CI
Muscle								
F NSW	18	1.16	1.36	4.52	0.55 ± 0.23	0.74	60.6	0.85–2.26
M NSW	25	2.06	1.83	7.26	0.74 ± 0.27	0.68	51.5	1.17–2.71
F QLD	45	3.79	4.23	15.32	1.34 ± 0.17	0.88	−28.9	3.14–6.68
M QLD	14	2.38	1.01	5.25	0.84 ± 0.25	0.77	−72.8	0.57–1.82
Dermis								
Ningaloo	20	1.61	1.7	5.04	0.22 ± 0.09	0.97	−6.4	1.00–2.51
GBR		1.42	1.54	23.33	1.21 ± 0.73	0.98	−18.9	0.89–2.70
Red Blood Cells								
Ningaloo	17	1.78	1.57	5.05	0.80 ± 0.69	0.98	2.7	0.99–2.69
Shark Bay	25	2.48	2.33	7.01	1.02 ± 0.93	0.95	−9.2	1.55–3. 47
GBR	14	2.58	2.33	5.61	0.89 ± 0.46	0.98	−7.4	1.30–4.08
Plasma								
Ningaloo	19	1.85	1.66	5.04	1.39 ± 0.69	0.93	−6.6	1.06–2.70
Shark Bay	45	5.65	5.48	23.33	0.93 ± 0.43	0.79	−3.6	3.97–7.38
GBR	14	6.72	5.93	15.07	1.09 ± 0.24	0.86	−12.9	3.30–10.40

The generalised linear model that included latitude, sex, total length and the interaction between latitude and sex had the highest support (wAICc = 0.76) and explained 65.3% of the deviance in the response (Table 3). This model indicated a shift to higher δ^13^C from smaller to larger sharks and towards lower latitudes for both sexes, trends that were more pronounced for females than males (Fig. 4B,C). In the analysis of the δ^15^N dataset, the four top-ranked models were all within two AICc points of the most parsimonious model (i.e. with the least number of parameters) including latitude only. The model indicated lower δ^15^N values towards lower latitudes, but explained only 7.6% of the deviance in the response.

**Table 3:** Ranked generalised linear models of δ^13^C and δ^15^N for each tissue.

Response	Model	df	AICc	Δ AICc	wAICc	%DE
Muscle						
**δ** ^**13**^ **C**	**lat + sex + TL + TL × lat**	**6**	**237.9**	**0.00**	**0.76**	**65.29**
δ^13^C	lat + sex + TL	5	241.4	3.57	0.13	63.01
δ^13^C	lat + sex + lat × sex	5	243.5	5.61	0.05	62.18
δ^13^C	lat + sex + TL + sex × TL	6	243.7	5.81	0.04	63.03
δ^13^C	lat + season + sex + TL	8	246.6	8.69	0.01	63.77
δ^13^C	lat + TL	4	247	9.13	0.00	59.74
**δ** ^**15**^ **N**	**lat**	**3**	**250.5**	**0.00**	**0.34**	**7.65**
δ^15^N	lat + sex + lat × sex	5	251.9	1.38	0.17	10.66
δ^15^N	lat + sex	4	252.6	2.10	0.12	7.74
δ^15^N	lat + TL	4	252.7	2.15	0.12	7.69
δ^15^N	lat + sex + TL	6	254.2	3.62	0.05	10.71
δ^15^N	lat + sex + TL + lat × sex	5	254.8	4.31	0.04	7.77
Dermis						
δ^13^C	location + TL	4	121.8	0.00	0.42	34.85
**δ** ^**13**^ **C**	**TL**	**3**	**122.7**	**0.86**	**0.28**	**27.93**
δ^13^C	location	3	123.8	1.96	0.16	25.54
δ^13^C	location + TL + TL × location	5	124.0	2.22	0.14	35.89
**δ** ^**15**^ **N**	**location**	**3**	**49.5**	**0.00**	**0.40**	**31.82**
δ^15^N	location + TL + TL × location	5	49.8	0.23	0.36	41.34
δ^15^N	location + TL	4	50.8	1.23	0.21	34.42
δ^15^N	TL	3	55.3	5.76	0.02	19.22
Red blood cells						
**δ** ^**13**^ **C**	**location + TL**	**5**	**212.9**	**0.00**	**0.86**	**55.62**
δ^13^C	location + TL + TL × location	7	216.6	3.73	0.13	56.72
δ^13^C	location	4	222.3	9.37	0.01	45.23
δ^13^C	TL	3	230.7	17.84	0.00	33.58
**δ** ^**15**^ **N**	**location**	**4**	**78.6**	**0.00**	**0.72**	**15.93**
δ^15^N	Location + TL	5	81.0	2.41	0.22	15.93
δ^15^N	TL	3	84.5	5.84	0.04	2.75
δ^15^N	location + TL + TL × location	7	85.9	7.25	0.02	16.37
Plasma						
**δ** ^**13**^ **C**	**location + TL**	**5**	**297.2**	**0.00**	**0.83**	**33.38**
δ^13^C	location + TL + TL × location	7	300.8	3.69	0.13	34.29
δ^13^C	location	4	303.3	6.16	0.04	25.76
δ^13^C	TL	3	307.2	10.06	0.00	19.70
**δ** ^**15**^ **N**	**location**	**4**	**218.2**	**0.00**	**0.66**	**8.66**
δ^15^N	location + TL	5	220.5	2.26	0.21	8.69
δ^15^N	TL	3	222.2	3.95	0.09	1.13
δ^15^N	location + TL + TL × location	7	224.3	6.04	0.03	9.83

The top six models for each response are shown; values in bold indicate the top ranked model (or the most parsimonious model). Degrees of freedom (df), sample corrected Akaike’s Information Criterion (AICc), change in AICc relative to the model with the lowest AICc value (ΔAICc), relative AICc weight (wAICc) and percent deviance explained (DE%). Lat = latitude, TL = total length (cm).

#### Dermis

Dermis was only collected from sharks captured at Ningaloo Reef and the GBR and data from both sexes were pooled for analysis due to low numbers of males in samples (n = 2). Samples from sharks on the GBR had greater dispersion over the δ^15^N axis (θ Ningaloo Reef = −6.4, θ GBR = −18.90) than those at Ningaloo (Fig. 4D). Although the size of the SEAb area was similar in both locations, niches were mostly distinct with sharks in GBR showing a larger TA and trophic diversity (Fig. 4D, Table 2) with only 0.2 probability of overlap. Of the three models selected to describe variation in δ^13^C values, the most parsimonious included only total length (wAICc = 0.28) and explained 28% of the deviance (Table 3). Model predictions indicated a shift to lower δ^13^C with increasing total length (TL) of sharks (Fig. 4E). For δ^15^N datasets, the three top-ranked models were within two AICc points, with the most parsimonious model including location only and explaining 32% of the deviance. Dermis tissue of sharks at Ningaloo Reef had higher δ^15^N than those of sharks from the GBR (Fig. 4F).

#### Red blood cells

Red blood cells were obtained from female sharks at Ningaloo and from both males and females at Shark Bay and on the GBR. Samples from different sexes were pooled within locations due to the very low numbers of males (3 in total). Plots of isotopic niches of sharks from all locations had ellipses stretched across the x-axis with high variation in δ^13^C values (Fig. 4G, Table 2). Sharks at Ningaloo Reef had a more distinct, smaller and less diverse niche than in Shark Bay and on the GBR based on SEAb, TA and CD (probability = 0.86 and 0.85, respectively), who were more similar to each other (Fig. 4G). In the analysis of the δ^13^C dataset there was one top-ranked model, which included total length and location and explained 56% of the deviance (Table 3). Red blood cells had lower δ^13^C with increasing size and higher δ^13^C at Shark Bay and on the GBR than at Ningaloo (Fig. 4H,I). The top-ranked model for the δ^15^N dataset only included location and explained 16% of the deviance, indicating marginally higher δ^15^N at Ningaloo (δ^15^N = 11.6 ± 0.1‰) in comparison to individuals from Shark Bay (δ^15^N = 11.4 ± 0.1‰) and the GBR (δ^15^N = 11.11 ± 0.12‰).

#### Plasma

Samples used for determination of niche area were collected from female sharks at Ningaloo Reef and from both sexes at Shark Bay and the GBR. Again, data were pooled for analysis between sexes within locations due to limited sample sizes. Sharks from the GBR and Shark Bay had significantly greater SEAb and TA areas than those at Ningaloo Reef (probability = 1 for both). However, sharks at Ningaloo Reef showed higher trophic diversity than in Shark Bay and on the GBR based on CD (Table 2). At all locations the dispersion of stable isotopic composition occurred on both axes (θ = −6.6 Ningaloo, θ = −3.6 Shark Bay, θ = −13.9 GBR, Table 2, Fig. 4J) and no niche overlap was evident in samples from Ningaloo Reef and the GBR, but there was an overlap of niche area with individuals from Shark Bay (0.30). The model including location and total length had majority support (wAICc = 0.83) and explained 33% of the deviance in the response (Table 3). The model predictions showed lower δ^13^C with increasing body length, with the lowest δ^13^C value recorded for sharks at Ningaloo Reef (Fig. 4K,L). For δ^15^N values, the top-ranked model only included location and had the majority of support (wAICc = 0.66), but explained only 9% of the deviance in the dataset.

### Diet stability

Six of the 18 tiger sharks tagged at Ningaloo Reef were considered to be residents as they were detected by receiver arrays at Ningaloo Reef (Table S1) for five or more months after tagging and tissue sampling. A mean Δδ^13^C value of −1.26‰ for the difference between red blood cell and plasma of resident sharks was considered as a threshold for the comparison of paired tissues that defined sharks as having stable diets. Due to a lack of tagging data from Shark Bay and the GBR in this study, the threshold calculated for Ningaloo was used in analyses for three locations.

Linear regressions between δ^13^C values from red blood cells and plasma from the same individuals had strong support when compared to a null model (wAICc = 1, 0.82 %DE) and the fitted line was then used for the identification of stability of diet (Figure S2a). The most parsimonious binomial generalised linear model (wAICc = 0.33, Table S2) to explain the probability of diet stability over one or two months only included location and explained 16% of the deviance. The predictions from this model showed that sharks at Ningaloo had a higher probability of having a stable diet than those sampled on the GBR and at Shark Bay (Figure S2b).

## Discussion

Our analysis of stable isotopes suggested that the functional role of tiger sharks varied among different marine habitats within the range of the species distribution along the tropical and temperate coasts of Australia. Typically, there was less variation in δ^15^N than δ^13^C values in slow turnover tissues (muscle, dermis and fin), indicating that the species remains close to the top of food webs at the body sizes we sampled, but that the ultimate sources of nutrition varied among localities and were dependent on body size, habitat and food-web composition.

Sharks sampled in the enclosed seagrass habitats of Shark Bay on the west coast and the large, shallow lagoon of the GBR showed the strongest evidence of long-term diets based in coastal-associated food-webs originating with seagrass. Dugong and turtles are likely prey in these localities^33, 41^. At higher latitudes, such as the coast of NSW, mean stable isotopic composition indicated a diet more closely linked to pelagic food chains. However, isotopic composition also appeared to be affected by an interaction between latitude and oceanographic setting. Most of the samples collected by QSCP were obtained from beaches south of the GBR, which are exposed to prevailing oceanic swells, upwelling and open waters across the shelf^42^. Stable isotopic compositions in this locality were similar to NSW, where equivalent oceanographic conditions prevail^43, 44^, but were also similar to samples from Reunion Island, an exposed and isolated island in the Indian Ocean that does not feature an extensive shelf or abundant seagrass^39^. In NSW, sharks were collected in deeper, pelagic waters at the edge of the continental shelf during gamefishing competitions. These results suggest that lower δ^13^C was not just a function of latitude but also the oceanographic setting and proximity of pelagic food chains. When plotted in isotopic space, values of δ^13^C of sharks sampled at Ningaloo Reef were between pelagic and seagrass food chains, as might be expected given that this location occurs at the narrowest point of the continental shelf along the Australian coast and so is strongly influenced by the Leeuwin Current, seasonal upwelling and the oceanographic conditions of the open ocean^45, 46^. Although seagrass beds exist at Ningaloo Reef in the shallow lagoon that extends one to two kilometres behind the reef crest to the shore^47^, these are relatively small in area compared to those in the inter-reefal lagoon of the GBR or at Shark Bay, where they are a dominant type of benthic habitat^48, 49^.

The average δ^15^N for tiger sharks in the present study differed greatly from those calculated by Hussey *et al*.^40^, who obtained samples from sharks caught in bather protection programs along beaches off the Natal coast of South Africa. Hussey *et al*.^40^ reported δ^13^C values for tiger sharks that were consistent with samples from the exposed sub-tropical coastlines of the present study (the QSCP and NSW datasets), but δ^15^N values were higher relative to all sampling locations in Australia. It is possible that the difference seen in South Africa may have been a reflection of differences in isotopic baselines and composition of the food-web, nutrient inputs or and/or sample treatment between these locations^50–52^.

Tiger sharks are a wide-ranging species and tracking studies show that these animals move between tropical and temperate environments and between coastal habitats and the open ocean off the continental shelf^35–38^. Our results suggest that the role of tiger sharks within food webs and their classification as an apex predator varies as they move across these environmental gradients. Tiger sharks occupied the apex position of the food-webs in seagrass habitats and shallow lagoons of Shark Bay and the GBR, where nearly all other shark species for which isotopic data were available had lower δ^15^N values. In contrast, at Ningaloo Reef, other, smaller sharks such as grey reef (*Carcharhinus amblyrhynchos*; adult size 1.9 m total length^53^) and blacktip reef (*C. melanopterus*; adult size 1.8 m total length^54^), had higher δ^15^N values^55^ than tiger sharks. These differences may reflect the feeding by reef sharks on mesopredator teleosts whose productivity is driven by complex coral reef food chains, whereas the diet of tiger sharks can also include species such as herbivorous teleosts, turtles and sirenians, which feed at a more basal level of the food web. As a result of the large home range areas of tiger sharks off the northwest coast of Australia^38^, it is also possible that sharks were feeding in different habitats or offshore areas (i.e. pelagic food-webs) with a lower stable isotopic baseline^56^, which could explain the differences between the stable isotopic composition of tiger sharks and the more resident reef shark community at Ningaloo Reef^57^.

In NSW, large oceanic fishes such as tunas and other sharks including makos (*Isurus oxyrinchus* and *I. paucus*)^58^ had higher δ^15^N values than tiger sharks, suggesting that they fed at higher trophic levels, which is supported by the high proportion of other predatory teleosts reported in the stomach contents of these species^59^. This was also the case for Australian fur-seals (*Arctocephalus pusillus doriferus*), which feeds on the continental shelf^60^. A lower trophic level for tiger sharks has also been reported in the coastal waters of warm temperate environments in southern Africa, where tiger sharks occupy the lowest trophic position within the community of large sharks^40^. Our results suggest that when tiger sharks move into pelagic and offshore habitats where other large teleost and sharks are present, these predators will occupy higher trophic levels than tiger sharks by having more specialised diets largely focused on fishes that are tertiary consumers or higher. Because no community isotopic data was available for the southern Queensland coast, it was not possible to determine the extent to which tiger sharks occupied the highest trophic level in these environments. However, the similarity of oceanographic conditions in this locality to the coast of NSW and the migrations of tiger sharks along much of the eastern coast of Australia^36^ suggests that they are likely to have similar trophic roles in both locations.

The position of tiger sharks in a food chain may also reflect sampling bias of other predators within these systems. For example, Vaudo & Heithaus^61^ and Heithaus *et al*.^41^ comprehensively sampled the fauna in shallow banks and sandflats habitats of Shark Bay and these shallow habitats might not be suitable for many large pelagic fishes such as tunas. The classification of tiger sharks as the highest trophic level predators in these habitats thus seems appropriate because all other species that might act in this role had been sampled. At Ningaloo Reef, sampling of the shark and predatory fish community was limited to reef environments, so that the larger pelagic species that might occupy higher trophic positions such as tunas could not be included in plots. With the exception of Shark Bay, where samples were collected over 6 years, there was also a lack of temporal context for sampling in Ningaloo and GBR localities. It may be possible that the diet and role of tiger sharks could vary across both seasons and years, as is the case with other marine predators^21, 23, 24^, although such variation may be limited, given the fact that the composition of samples in terms of δ^15^N values was very similar across all locations and that the mean stable isotopic composition from muscle tissues of tiger sharks analysed by earlier studies on the GBR^68^ were nearly identical to those presented here.

There was some evidence that size affected the diets of tiger sharks, although patterns were inconsistent among tissues. Muscle, a slow turnover tissue, provided the largest dataset available for analysis and showed higher δ^13^C with increasing size (total length) suggesting a diet increasingly shifted towards prey from seagrass habitats. This is likely due to larger sharks having a greater capability to attack and subdue large marine herbivores such as turtles and dugong than smaller, younger sharks. It is also consistent with the results of studies of the stomach contents of tiger sharks that show a change in diet towards larger prey with increasing size^32^. There was also a shift to higher δ^13^C at lower latitudes where these large herbivores such as turtles and dugongs are more abundant. In addition, plots of isotopic niches calculated from muscle tissues and generalised linear models suggested that there was some effect of sex on diet. Female sharks had a larger trophic niche (SEAb, TA and CD) and showed greater variability in δ^13^C values, a pattern that may be due to high physiological and energetic demands associated with reproductive cycles^63^ which may require a more adaptable and opportunistic diet than male sharks. However, analysis of dermis tissue revealed an opposite pattern to muscle, with decreasing δ^13^C values with increasing body size. This result must be treated with caution because sample sizes of dermis were low (n = 34) and the range in total lengths of the animals from which they were obtained were truncated compared to analyses of muscle. Furthermore, with the exception of a single individual, dermis samples of the largest sharks (>350 cm TL) were only collected from Ningaloo Reef and these clustered at one end of the plot, thus may have strongly weighted the relationship with size. Plots of the niche areas for all tissues did, however, separate samples from Ningaloo Reef from those collected on the GBR, suggesting a diet shifted towards more planktonic food webs at the former locality.

Analysis of fast-turnover tissues (red blood cells and plasma) also displayed a decrease in δ^13^C values with size, contrasting with results for muscle. In this case the shift in the relationship with size could represent a seasonal or short term (months) component to the diet that differed between smaller and larger sharks. Given that blood samples were only taken within a time window of a few months of the year, even where sampling extended over multiple years (as was the case at Shark Bay), it is difficult to determine how often this trend with size would be likely to occur. Notably, the effect of location evident from the analysis of muscle tissue was also present in these tissues, with the trophic niches of sharks from the GBR and Shark Bay largely overlapping and sharks at Ningaloo having a mostly separated trophic niche with lower δ^13^C values. The analysis of plasma suggested that sharks sampled in Shark Bay and the GBR had much wider trophic niches than those at Ningaloo, consistent with the stability analysis that also suggested that diets of sharks at Ningaloo were more stable in short term (weeks-months) than those of sharks on the GBR and at Shark Bay. The low probability of sharks showing a stable diet in Shark Bay and the GBR suggests high variability in diet across these short temporal scales and supports the idea that tissues with slower rates of turnover (muscle, dermis, fin) may provide a better picture of the overall diet of tiger sharks. Nevertheless, results from the analysis of diet stability should be interpreted with caution as residency was defined on the basis tracking data that was only collected from sharks at Ningaloo Reef.

Overall, the local environment appeared to be a better determinant of the diet of tiger sharks than latitude, despite the ability of the species to migrate long distances from the tropics to cool temperate environments^36, 38^. Sharks sampled at locations that were separated by <300 km such as Ningaloo and Shark Bay displayed very distinct stable isotopic compositions, which is unexpected considering the movement patterns of these sharks between those locations^38, 64^. In contrast, stable isotopic composition of sharks sampled from the mid-NSW coast were similar to those collected in central and southern QLD, around 1000 km to the north, which may reflect seasonal movements along that coastline^36^. The isotopic composition of sharks in more temperate and pelagic habitats in Australia were also similar to sharks sampled at Reunion Island^39^, an isolated atoll in the far western Indian Ocean. Environment also appeared to determine the degree to which the species acted as an apex predator, although in some localities (notably Ningaloo Reef and southern Queensland) more comprehensive sampling of other elements of pelagic consumer fauna is required to accurately place tiger sharks within the trophic pyramid.

The description of the species as a generalist predator^22, 31, 32, 39^ appears justified with stable isotopic compositions being habitat-dependent and ranging from seagrass (Shark Bay) to planktonic food chains (NSW coast) or a mixture of both (Ningaloo Reef). Given that individuals tagged at Shark Bay and Ningaloo have dispersed across the north-western coast of Australia^36, 38^ and sharks tagged in NSW have dispersed to the tropical Queensland coast^36^, it appears that the degree of specialisation by an individual shark on prey at any one time is likely to be context-dependent and reflect both resource availability and to some extent, body size. Although tiger sharks at Ningaloo Reef showed greater trophic diversity based on tissues with fast turnover (e.g. plasma), they also had a greater stability of diet than sharks in Shark Bay and on the GBR. This might suggest that tiger sharks at Ningaloo Reef have wider diet than those in Shark Bay and on the GBR, but are more likely to remain within the same habitat, at least over short time frames (weeks – months).

Our results emphasise the flexibility of the trophic ecology and role of tiger sharks throughout their range in Australia. Generalist predators such as tiger sharks are able to explore multiple habitats and food-webs, likely adapting foraging strategies to specific prey in different habitats. This generalist behaviour may both drive or be driven by scale- and habitat-dependent space use by the species. However, separating the cause and consequence of the relationship between movement and trophic ecology for large, highly mobile, top-level predators is still challenging^4^. An important next step to better understand the feeding behaviour of these sharks is offered through the combination of diet analysis and the information collected by satellite tags that could provide a more comprehensive picture of animal movement behaviours and patterns of prey search that can then be matched against animal trophic ecology.

## Methods

### Sample collection

Samples were collected under permit numbers: 2563 (WA Department of Fisheries), SF010311 (WA Department of Parks and Wildlife); G14/36467.1 and G14/37133.1 (Great Barrier Reef Marine Park Authority); 100541, 165491 and 56095 (NSW Department of Primary Industries and Fisheries); QS2009/GS001, QS2010/MAN26 and QS2010/GS059 (QLD Department of Environment and Resource Management). All methods and experimental protocols were performed in accordance with approved guidelines by Animal Ethics Committees from the University of Western Australia (RA/3/100/1209), James Cook University (A1974) and University of Queensland (CMS/300/08/DPI/SEAWORLD and CMS/326/11/DPI).

Tiger sharks were sampled from Ningaloo Reef and Shark Bay in Western Australia, the Great Barrier Reef (GBR), and the coast of Queensland and deeper continental shelf waters (>100 m) of New South Wales (NSW) (Fig. 1). Two different datasets were available from Queensland, one composed of muscle samples collected between 2005 and 2014 from sharks captured by the Queensland Shark Control Program (QSCP) between latitudes of 28°S and 10°S (hereafter the QSCP dataset), and another of multiple tissues (muscle, dermis, blood) from individual sharks collected at different locations throughout the Great Barrier Reef (hereafter the GBR dataset) (Fig. 1). The majority of the individuals sampled in the QSCP were captured in coastal waters of southern Queensland, below the latitude of the southern extremity of the GBR. QSCP and GBR datasets were not pooled due to the different analytical methods applied to each (see below).

Sample collection was a collaborative effort across multiple research programs in Australia and as a consequence, the number of samples for each location, sex, types of tissue collected and sampling methods varied among locations. For the sampling at Ningaloo Reef and the GBR, tiger sharks were caught using drumlines inside the reef lagoon and led onto a submerged platform. The platform was raised clear of the water and the shark was restrained by a tail rope. A hose with a continuous flow of oxygenated seawater was inserted in the mouth of the animal. The shark was then measured and sexed and fitted with electronic tags prior to release (described below). A small sample (3 g) of white dorsal muscle and dermis were collected with a scalpel or an 8-mm diameter tissue punch, about 5 cm lateral to the first dorsal fin. Blood samples of 5 ml were collected with an 18-gauge needle from the caudal vein. The blood was separated into plasma and red blood cells with a portable centrifuge (6500 rpm) and all samples were immediately frozen in either liquid nitrogen (−80 °C) or −20 °C freezers after collection. Dermal denticles were removed from dermal samples prior to analysis. Stable isotope data from sharks captured in Shark Bay were collected by a multi-year program^30, 41^ and added to the dataset. For field and sampling procedures for sharks captured in Shark Bay see Heithaus^33^ and Wirsing *et al*.^62^. For sharks caught by the QSCP, muscle samples were collected from the musculature adjacent to frozen vertebrae. In NSW, shark muscle was collected from the dorsal region next to the first dorsal fin at recreational game-fishing competitions and frozen immediately for storage. Sampling procedures for Queensland and NSW are described by Holmes *et al*.^65^.

### Stable isotope analysis

Samples from Ningaloo Reef, the GBR, the QSCP and NSW were analysed by the Western Australian Biogeochemistry Centre at the University of Western Australia. Samples were freeze dried for 48 h and homogenised using a Mixer Mill MM 200 with 6.4-mm ball bearings. Samples were analysed using a continuous flow system in a Delta V Plus mass spectrometer coupled to a Thermo Flush 1112 via Conflo IV (Thermo-Finnigan/Germany). Stable isotope ratios were expressed as δ^13^C and δ^15^N and reported in parts per mil (‰) relative to the standard reference Vienna Pee Dee Belemnite (VPDB) for δ^13^C and atmospheric N_2_ for δ^15^N. Samples were standardised against primary analytical standards from the International Atomic Energy Agency, Vienna (δ^13^C: NBS22, USGS24, NBS19, LSVEC; δ^15^N: N1, N2, USGS32 and laboratory standards). The analytical precision (standard deviation of mean values) of both carbon and nitrogen isotopes was 0.1‰. Samples had mean C:N ratios of 2.5 ± 0.5 (mean ± SD) for samples analysed at the Western Australian Biogeochemistry Centre, and because elasmobranchs are reported to have low lipid content with relatively small changes to δ^13^C values after lipid extraction, we did not correct δ^13^C values for the effects of lipids^41, 55, 61, 66, 67^. Samples from Shark Bay were analysed at Yale University and Florida International University with analytical precision of 0.1–0.2‰ for δ13C and 0.02–0.1‰ for δ^15^N and showed low C:N ratios (2.4 ± 0.4).

### Data analysis

#### Stable isotopic composition

In order to assess variations in stable isotopic composition of tiger sharks among widely distributed locations, δ^15^N and δ^13^C values of muscle samples previously described by studies in South Africa^40^, the Great Barrier Reef^68^ and Reunion Island^39^ were plotted against the average δ^15^N and δ^13^C values of slow and intermediate turnover tissues (dermis, muscle, and fin) from this study. Dermis is likely to have faster turnover rates than muscle^23, 69^, however, in the absence of muscle samples at Ningaloo, dermis was considered as the longer-term representation of diet. Dermis is a poorly vascularised structural tissue that may reflect longer-term (months) signals in diet^70^ when compared to tissues that are metabolically active such as blood^19^. To assess the trophic position of tiger sharks in each ecosystem, the stable isotope composition of each tissue (muscle, dermis, red blood cells and plasma) for all sharks at each location was also plotted against that of taxa from different trophic positions from each location (Ningaloo Reef, Shark Bay, GBR and NSW), with values extracted from published literature. Additional isotope data from muscle tissue of four tiger sharks sampled on the GBR (Lizard Island and southern GBR) between 2013 and 2014, and analysed at James Cook University, were combined with isotopic values from the literature for the comparison with the values obtained in the present study. As earlier studies were analysed in different laboratories with varying sample handling and processing protocols^71^, these comparisons were made visually and no statistical analysis was attempted. Conversion corrections between stable isotope values of muscle and dermis were not calculated since trophic discrimination factors for dermis have not been defined^69^.

#### Isotopic niche

Isotopic niche space occupied by tiger sharks in each location in Australia was measured by calculating standard ellipse areas corrected for sample size (SEAc), the SEAc parameters of eccentricity (E) and theta (θ), the Bayesian estimation of SEA (SEA*b*) and the overlap between paired groups as a probability index with values between 1 and 0^72^. The Bayesian approach used vague and non-informative priors and was built with 2 Markov chains with 20,000 steps per chain, a discarded burn-in of 1,000 iterations and a thinning interval of 10. This produced a range of SEAb with 95% credible intervals that allowed for direct probabilistic interpretation of pairwise comparisons between groups that could be interpreted as the probability of the Bayesian ellipse area of one group being larger than another^72, 73^. The total area (TA) occupied was calculated as the areas of the convex hull that incorporated all individuals and represents a measure of niche width and reflects the isotopic diversity of a group^61, 74^. The mean distance to centroid (CD) was calculated as the distance in isotopic space of each individual to the mean of all individuals and represented a measure of the average trophic diversity within a group^61^. As eccentricity (E) was related to the variance on the x (δ^13^C) and y (δ^15^N) axes and to the circularity of the SEAc, high values of E were representative of ellipses that were stretched either in the x or y-axes. Theta was a measure of the inclination of the ellipse and is reported here in an angular range between −90 to 90, with positive and negative values indicating the direction of inclination of standard ellipses^72, 73^. Values for each tissue were analysed separately using the R packages *siar* ^75^ and *SIBER* ^76^. Sexes were analysed as separate groups within a region for datasets that contained a representative number of samples (n > 10) for both male and female sharks.

#### Spatial patterns of isotopic composition of multiple tissues

Generalised linear models (GLMs) with a Gaussian distribution were used to assess if δ^13^C and δ^15^N varied spatially (by location or latitude), and in relation to sex and size of sharks and at different temporal scales by using tissues with different turnover rates. Data from each tissue (muscle, dermis, red blood cells and plasma) was modelled separately using both δ^13^C and δ^15^N as response variables and a set of explanatory variables that were dependent on the data available for each tissue. The set of predictors varied among tissues and was selected based on datasets available (e.g. sex was only used as predictor if the number of males was sufficient, whereas season was only included in the analysis of datasets of muscle). For muscle, the stable isotope composition was analysed with regard to the latitude, season (austral summer, autumn, winter and spring), sex, total length (TL, in cm) and interaction terms between sex and latitude, and sex and TL. For dermis, stable isotopes were assessed against the categorical variable (location) rather than the continuous variable (latitude), due to sampling locations on the west and east coast of Australia having similar latitudes. Total length and an interaction term between location and TL were also included. For red blood cells and plasma, variables analysed included location, TL, and the interaction terms between TL and location. Fin samples were only available from Shark Bay and, therefore, these were not included in this analysis. All potential combinations of these predictor variables were fit with the package *MUMIn* ^77^. Model selection was done by ranking the models by Akaike’s Information Criterion corrected for sample size (AICc) and the AICc weight (wAICc) which varies from 0 (no support) to 1 (complete support). When multiple models were ranked equally (models within 2 AICc units), the most parsimonious model was selected (the model containing the lowest number of explanatory variables). Goodness of fit was assessed by the percentage deviance explained (%DE). Conditional plots for each variable in the top-ranked model were plotted using the R package *ggplot2* ^78^ and *visreg* ^79^.

#### Diet stability

Tiger sharks (18 individuals) at Ningaloo Reef were also tagged internally with a V16TP or V16 acoustic transmitters (Vemco Ltd., Canada) (Table S1). Upon capture, sharks were restrained and rolled to expose their ventral side. A 4 cm incision was made along the centre line of the abdomen, an acoustic tag was inserted and the incision was closed with three stiches in the muscle and skin using surgical sutures. Sharks tagged with an acoustic transmitter were monitored by receiver arrays around Ningaloo Reef (Figure S1) deployed by the The IMOS animal tracking facility. This tracking allowed sharks that were resident at Ningaloo Reef to be identified so that their isotopic composition could be used as a baseline value for analyses. An earlier study at Ningaloo Reef demonstrated that tiger sharks either resided in the areas where they were tagged for up to 6 months or departed and ranged 1000s of km along the coast of Western Australia^38^. Consequently, we expected that individuals that remained resident after tagging and tissue sampling were likely to have been resident to Ningaloo Reef prior to capture. As receiver downloads were sporadic during the duration of the study, sharks were defined as residents if they were detected by the receiver arrays for at least five successive months after tagging and prior to the last download of receivers (Table S1), again consistent with residency times recorded by earlier studies^38^. As blood has an isotopic turnover rate of months^80^, the stable isotopic composition of this tissue was used as a baseline for resident sharks.

Stability of diet was assessed based on differences in δ^13^C between paired tissues (red blood cells and plasma) for the locations where multiple tissues were collected from the same sharks (Ningaloo Reef, Shark Bay and GBR). Plasma has a faster isotopic turnover rate than red blood cells (a half-life of 32 vs. 60 days)^19^. The contrasting turnover rates of these tissues allowed for comparison of isotopic values within an individual, giving an insight into the temporal stability of diet^22, 81^. Preliminary analysis of this dataset and previous studies of tiger sharks in Shark Bay showed greater inter-tissue differences in δ^13^C than in δ^15^N^22, 82^, therefore, this analysis only considered δ^13^C values. Linear regression was fitted to the paired values of δ^13^C for sharks from Ningaloo Reef, Shark Bay and the GBR in order to describe the consistency of differences in δ^13^C between tissues and to quantify the inter-tissue relationship, as a measure of tissue discrimination differences^82–84^. As paired tissue discrimination shows stable relationships in other species including seabirds, mako (*Isurus oxyrinchus*) and white sharks (*Carcharodon carcharias*)^81, 85^, it can be used to compare stability of diet in samples from various locations by identifying individuals that are not in a steady state. wAIC values were compared for the regression of δ^13^C between red blood cells and plasma against a null model. If there was support for the model over the intercept only model (null) and the R^2^ value was high (>0.8), the relationship was considered significant and stable. Stable relationships between Δδ^13^C values of paired tissues suggest a stable discrimination of differences among locations and validate use of the fitted line of the regression and δ^13^C differences to investigate dietary stability in individual sharks. Since only a relationship with strong support was used among different locations, mean values of the paired tissue differences of resident sharks at Ningaloo Reef were used as a proxy for the δ^13^C for discrimination between red blood cells and plasma (Δδ^13^C). Paired tissue differences for each individual (Δδ^13^C) were then plotted over the fitted line and interval (±mean Δδ^13^C) around it. Data points that plotted more than Δδ^13^C above the fitted line indicated plasma values with higher δ^13^C values than predicted by the model and data points that plotted less than Δδ^13^C below the fitted line indicated plasma values with lower δ^13^C than predicted by the model^83, 84^. Values of plasma outside the ±mean Δδ^13^C interval area around the fitted line, therefore, indicated a recent shift in diet. Values within the ±mean Δδ^13^C interval around the fitted line were considered as “stable” and were assigned a value of 1. Values that were either higher or lower than the ±mean Δδ^13^C interval were considered “non-stable” and assigned a value of 0. Generalised linear models with a binomial distribution were used to test the probability of sharks having a stable diet in relation to total length and location. Models containing all potential combinations of the predictor variables (location, total length and an interaction term with total length and location) were fitted and model selection was as described previously.

## Electronic supplementary material

Supplementary information
