# Supplementary material for: The trophic role of a large marine predator, the tiger shark Galeocerdo cuvier
Source: Sci Rep. 2017 Aug 9;7:7641. doi: 10.1038/s41598-017-07751-2 (PMC5550416; doi:10.1038/s41598-017-07751-2)
Supplement: Supplementary file 1 — Supplementary information [file 41598_2017_7751_MOESM1_ESM.pdf]

Manuscript title: The trophic role of a large marine predator, the tiger shark *Galeocerdo cuvier*

Author list: Luciana C. Ferreira, Michele Thums, Michael R. Heithaus, Adam Barnett, Katya Abrantes, Bonnie J. Holmes, Lara M. Zamora, Ashley J. Frisch, Julian G. Pepperell, Derek Burkholder, Jeremy Vaudo, Robert Nowicki, Jessica Meeuwig and Mark G. Meekan

Supporting information

Table S1 Details of tiger sharks tagged at Ningaloo Reef in April-May 2015, their duration of detection (until last receiver download) by receivers stations at Ningaloo and their behavioural classification (resident or non-resident).

Shark ID	Sex	TL (cm)	Monitoring (months)	Behaviour
1	F	373	3	Non-resident
2	F	319	1	Non-resident
3	F	372	2	Non-resident
4	F	391	1	Non-resident
5	F	407	2	Non-resident
6	F	285	6	Resident
7	F	363	0	Non-resident
8	F	304	5	Resident
9	F	390	6	Resident
10	F	390	3	Non-resident
11	F	303	3	Non-resident
12	F	405	0	Non-resident
13	F	322	5	Resident
14	F	352	0	Non-resident
15	F	268	5	Resident
16	F	290	0	Non-resident
17	F	393	1	Non-resident
18	F	282	6	Resident

Table S2. Ranked binomial generalised linear models of stability of diet for sharks (stable with the local food web), using paired tissue values of $\delta^{13}C$ from red blood cells (RBC)-plasma comparisons. All models are shown, and values in bold indicate the most parsimonious model. Sample corrected Akaike's Information Criterion (AICc), change in AICc relative to the model with the lowest AICc value (ΔAICc), relative AICc weight (wAICc) and percent deviance explained (DE%). TL= total length (cm)

Response	Model	df	AICc	Δ AICc	wAICc	%DE
**Stability**	**location**	**3**	**74.8**	**0.00**	**0.61**	**9.87**
Stability	Location + TL	4	76.2	1.40	0.30	11.10
Stability	TL	2	79.9	5.11	0.05	0.17
Stability	Location + TL + TL × location	6	80.1	5.28	0.04	12.51

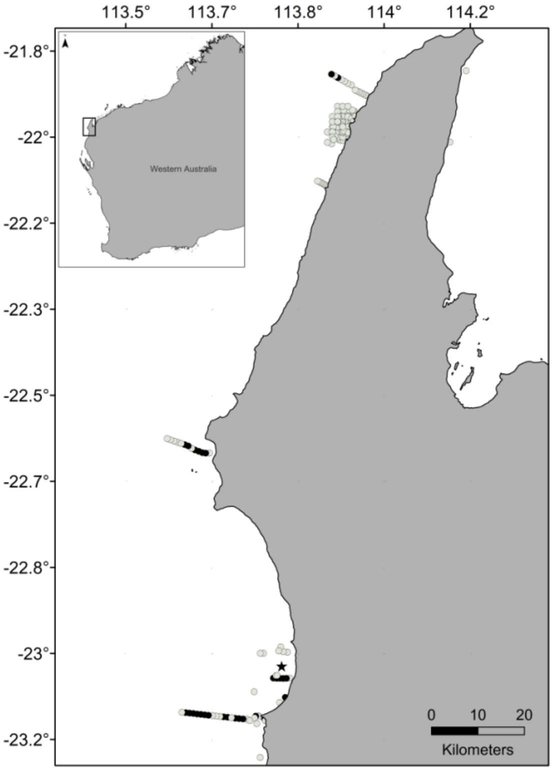

Figure S1. Location of acoustic receivers (grey and black circles) around Ningaloo Reef, Australia. Black circles represent receivers where tiger sharks were detected. Star indicates tagging location. Map was created with ArcGis 10.3 (http://www.esri.com/).

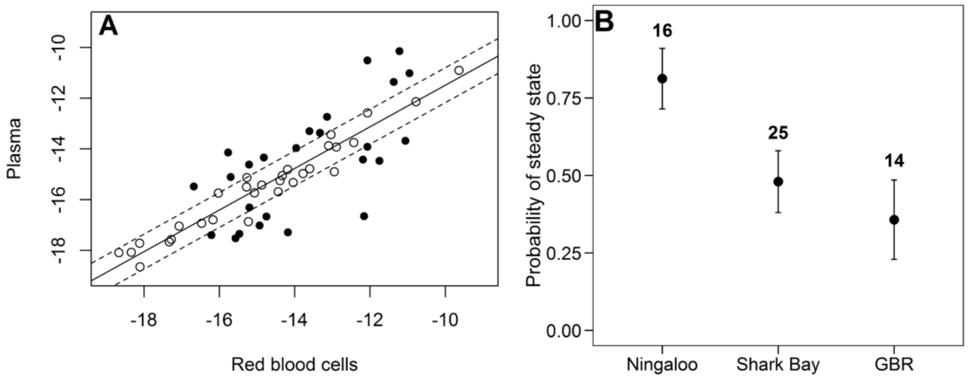

Figure S2. Relationship between δ13C values of paired tissues of fast (plasma) and intermediate (red blood cells) turnover rates (A). Individuals with a difference between tissues (calculated from resident sharks) larger than mean differences Δ13C are highlighted in filled symbols. Open symbols represent individuals in steady state (stable). Solid line represents the relationship between paired tissues and hashed lines enclose the area within ± mean Δ13C. Partial dependence plots for the relationship between the stability of diet (probability of steady state) and the explanatory variable in the top-ranked model; location (B). Numbers indicate sample sizes.
